# Dyadic Coping in Aging: Linking Self-Perceptions of Aging to Depression

**DOI:** 10.3390/geriatrics9060147

**Published:** 2024-11-11

**Authors:** Jose Adrián Fernandes-Pires, Guy Bodenmann, María Márquez-González, María del Sequeros Pedroso-Chaparro, Isabel Cabrera, Laura García-García, Andrés Losada-Baltar

**Affiliations:** 1 Department of Psychology, Universidad Rey Juan Carlos, 28922 Madrid, Spain; jose.fernandes@urjc.es (J.A.F.-P.); laura.ggarcia@urjc.es (L.G.-G.); 2 Department of Psychology Clinical Psychology for Children/Adolescents and Couples/Families, University of Zurich, 8050 Zurich, Switzerland; guy.bodenmann@psychologie.uzh.ch; 3 Department of Biological and Health Psychology, Universidad Autónoma de Madrid, 28049 Madrid, Spain; maria.marquez@uam.es (M.M.-G.); mariadelsequeros.pedroso@udima.es (M.d.S.P.-C.); i.cabrera@uam.es (I.C.); 4 Department of Psychology, Universidad a Distancia de Madrid, 28400 Madrid, Spain

**Keywords:** dyadic coping, negative self-perceptions of aging, depressive symptoms, middle-aged and older adults

## Abstract

Negative self-perceptions of aging have been linked to poorer health and quality of life and predict significantly depressive symptomatology. The support provided by the partner may have an impact on the effects of self-perceptions of aging on depressive symptoms; a close relationship can go along with additional stress or resources and benefits. The present study analyzes the relationship between negative self-stereotypes and depressive symptomatology, considering positive and negative dyadic coping (DC) as moderator variables in this association. **Method:** Participants were 365 individuals (convenience sample) 40 years or older (M = 60.86) involved in a partner relationship. Participants completed a questionnaire that included the following variables: negative self-perceptions of aging, positive DC (e.g., “My partner shows empathy and understanding to me”), negative DC (e.g., “When I am stressed, my partner tends to withdraw”), and depressive symptomatology. Two moderation models were tested by linear regression. **Results:** The effect of negative self-perceptions of aging on depressive symptoms was moderated by positive and negative DC only in women. The effect of negative self-perceptions of aging appears to be smaller among those women with higher levels of positive DC and lower levels of negative DC. **Conclusions:** Positive DC might buffer the association between negative self-perceptions of aging and depressive symptoms. Negative DC might amplify this association, as it is associated with lower well-being among women who express negative self-perceptions of aging. **Implications:** Training couples in strategies for providing supportive dyadic coping may be a resource to buffer the negative effect of negative self-perceptions of aging on well-being.

## 1. Introduction

Negative stereotypes about aging are a global phenomenon, influencing people’s behavior and well-being when they come either from others or from oneself. Prolonged exposure to negative attitudes toward aging from childhood to adulthood results in the development of negative perceptions of aging and accepting of age stereotypes [1]. According to Levy’s stereotype embodiment model [1,2], it is during middle age and older adulthood that negative experiences related to aging—such as beliefs that aging inevitably leads to cognitive decline, disability, and dependence on others [3] have already been internalized and can operate unconsciously. As people advance into older adulthood, these stereotypes become self-relevant, fostering negative self-perceptions of aging. At the point when stereotypes about aging are directed toward oneself, they transform into negative self-perceptions that become part of the individual’s core beliefs [2].

When negative aging stereotypes are activated by experiences congruent with aging-related attributions, such as health decline or loss of independence, they can result in negative cognitive–affective cycles, leading individuals to fear aging [4]. As a consequence, the individual may stop seeking help or reject support, attributing their mood or everyday problems to aging or the aging process, and believing that their situation is unchangeable. These negative self-perceptions of aging can influence outcomes through behavioral, physiological, and psychological pathways, potentially impacting mental health [1].

Longitudinal studies have found negative self-perceptions of aging to predict depressive and anxiety symptoms [5]. In most studies on the topic, these negative perceptions have been linked to poorer longevity and quality of life [5,6,7]. In addition, negative self-perceptions of aging have been reported to be associated with risky health behaviors such as unhealthy diet, medication noncompliance, and harmful drinking and smoking [5]. Negative elf-perceptions of aging have been associated with poorer physical health [5,8,9], and both variables are considered vulnerability factors in the development of depressive symptoms [5,10,11]. Research indicates that individuals with negative self-perceptions of aging are more susceptible to stress. Specifically, they are more likely to encounter stressful health events and exhibit stronger reactions to stressors whether in controlled lab settings or everyday life [1,12,13].

Although social context is known to impact perceptions of aging, research exploring the relationship between self-perceptions of aging and close social relationships remains limited [14]. Studies have shown that supportive romantic relationships can lead to more positive self-perceptions of aging, while relationship tension correlates with negative self-perceptions of aging [15,16,17,18,19]. In some cases, these associations may vary by gender. Kim et al. found that receiving support from a partner tends to reduce anxiety about aging in women, while in men, relationship tensions appear to have a greater impact, potentially exacerbating negative feelings about aging [17,18]. Although moderating effects of spousal support and strain are noted in the domains of impairment, physical health, and subjective age [18,20,21], the potential moderating role when negative self-perceptions of aging are already internalized has not been studied. It is unknown how perception of support might amplify or dampen the effects of negative self-perceptions of aging on depressive symptoms.

Research is consistent in showing the protective effect of close relationships on emotional well-being [22,23]. Positive partner relationships seem to be linked to significant benefits [23,24,25]. However, it has been found that marital discord predicts depressive symptoms [26]. These associations can be examined within the context of the Systemic Transactional Model (STM; [22,25]), which posits that an individual relies on both personal resources (individual coping) and those of their partner (dyadic coping, or DC) to manage stressful events. According to the STM, individuals in supportive close relationships may benefit from additional resources through problem-focused and emotion-focused support. Different forms of dyadic coping are distinguished, and these forms can be either emotion-oriented or problem-oriented, and they can be of a positive or negative nature. Positive and negative dyadic coping are conceptualized as distinct but related processes, not as direct opposites [24,27]. Positive DC involves supportive behaviors that help reduce stress reactions, while negative DC refers to maladaptive behaviors such as hostility, ambivalence, or superficial support, which can potentially exacerbate stress responses [28,29]. One or both forms of coping may occur within the same relationship at different times or in response to different stressors, reflecting the complexity of relational dynamics [28,29].

When individuals hold negative self-perceptions of aging, these cognitions may increase sensitivity to potential stressors [12,13]. However, perceiving that they have adequate support from their partner could mitigate the impact of such a stress response. Conversely, believing that they have negative support could exacerbate the effects of stress reactions facilitated by negative self-perceptions of aging. Thus, this model assumes that positive DC might mitigate the negative effects of negative self-perceptions of aging on well-being, by the mechanism of moderation, while negative DC can amplify this association and be related to lower well-being in persons with negative self-perceptions of aging.

Although adaptive DC is beneficial across all ages and gender [30], we focused on middle-aged and older adults because self-perceptions of aging become more pronounced and personally relevant during these life stages [1]. In late adulthood, individuals encounter significant age-related challenges including health, autonomy, and relational goals [30]. Furthermore, older adults often experience reduced social support outside their marriages [31], which makes spousal support increasingly important. Previous studies have suggested that both husbands and wives are equally capable of displaying supportive coping behaviors to their partners [27,32]; research suggests that dyadic coping may have different effects depending on gender. Although positive DC has a positive effect on both genders, several studies have shown that the effect of dyadic coping seemed to be more important for women than for men [33,34,35]. Consequently, not perceiving or receiving optimal support from their partners, especially when these women exhibit stress from a negative situation, can contribute to them feeling worse about themselves [36].

Summarizing the findings described above, it seems that the support received from the partner may influence the consequences that negative self-perceptions about aging have on older adults’ distress by either reducing its impact (through positive coping) or increasing it (through negative coping). The present study is aimed at analyzing the relationship between negative self-stereotypes and depressive symptomatology, considering the individual’s perception of support from their partner (DC by the partner) as a moderating variable in this association. Variables that have been related in previous studies to the main variables of the study have also been considered, such as age, gender, level of education, relationship duration, having offspring, and physical health [5,9,17,18,30,33]. This evaluation focuses on the perceived support reported by only one member of the couple. Including DC in a model studying the impact of negative self-perceptions of aging on well-being (i.e., depression) might be particularly promising, as it may shed light on the interpersonal factors potentially shaping this process. Furthermore, we will explore these associations by gender. There are three specific hypotheses. (a) Higher negative self-perceptions of aging will be associated with more depressive symptomatology. (b) There will be a statistically significant moderation effect of DC in the association between self-perceptions of aging and depressive symptoms. This moderation will show a buffering effect in the case of positive DC and an increase in the size of the association between negative self-perceptions of aging and depressive symptoms in the case of negative DC. (c) Since no previous research suggests a stronger moderating effect for either men or women, the findings of this study regarding gender will be exploratory.

## 2. Method

### 2.1. Participants and Procedure

The sample consisted of 365 white European individuals residing in Spain. To participate in the study, individuals had to be at least 40 years old, not show any explicit cognitive or functional impairment that would prevent them from attending to and answering the research protocol, and be in a marital/partner relationship of at least one year’s duration. As shown in Table 1, the sample was composed mostly of female participants (59.3%) and had a mean age of 60.86 years (SD = 10.66; range = 40–90). The average duration years of the relationship was 31.69 years (SD = 14.60; range = 1–70).

The recruitment process was conducted through social media, snowball sampling, and collaboration from cultural centers, associations, and institutions that have regularly worked with the research team. Data collection took place from January 2021 to July 2022, and assessments were conducted through face-to-face interviews (67.8%) or an online survey (32.2%). The characteristics of both groups can be seen in Supplementary Table S1. In addition, the analyses included in this study were repeated controlling for type of assessment (see Supplementary Materials). As no differences were observed in the obtained associations with respect to the models without controlling for this variable, we opted not to include type of assessment in the model. Participants were asked to report whether their partner would also participate in the study. In those cases, the birth date of each member of the couple was requested so that data from each couple could be afterwards matched. Respondents were asked to fill out the questionnaire independently of their partners. Data were collected from individuals, rather than from couples, and are thus analyzed at the individual level. All participants provided their consent to participate in the study, which was approved by the Ethics Committee of the Universidad Rey Juan Carlos.

### 2.2. Variables and Instruments

The following sociodemographic variables were assessed: gender, age, years of marriage/partner relationship, education level, and having offspring. The educational level had seven possible answers, ranging from 1 (no formal education, 0 years of education) to 7 (master’s degree, doctorate, or post-doc, 19 years of education). Additionally, respondents were asked if they had offspring with two options: Yes (1) or No (2).

Physical health. Physical health was assessed using the Spanish version [37] of the Physical Functioning subscale from the Short Form 36 Health Survey (SF-36, [38]). This subscale consists of 10 items (e.g., “Does your health now limit you in walking more than a mile?”), measuring physical functioning and limitations. Responses range from 1 (no, not limited at all) to 3 (yes, limited a lot). Higher scores indicate greater limitations in physical functioning. In the present study, the Cronbach’s alpha for this subscale was 0.88 (0.91 for men, 0.85 for women).

Self-perceptions of aging. Self-perceptions of aging were assessed through the Subjective Aging Perception Scale from De Garcia [39]. This scale captures individuals’ attitudes toward their own aging process. It consists of twelve items (e.g., “I think I am pretty fit for my age,” “I think I have the same mental agility as before”). In order to facilitate interpretation, scores were reversed so that higher scores meant more negative self-perceptions of aging. Cronbach’s alpha for this scale in the present study was 0.81. (0.81 men, 0.81 women).

Dyadic Coping. Dyadic coping was assessed with the subscales of positive and negative dyadic coping perceived in the partner from the Dyadic Coping Inventory [40]. This scale focused on the perceived support reported by only one member of the couple. The positive dyadic coping (positive DC) subscale is composed of five items (e.g., “My partner helps me analyze the situation so that I can better face the problem”), and the negative dyadic coping (negative DC) subscale is composed of four items (e.g., “My partner provides support, but does so unwillingly and unmotivated”), with a 5-point Likert-type scale from 1 (very rarely) to 5 (very often), where higher scores indicate more positive or negative dyadic coping. The internal consistency (Cronbach’s alpha) of the scale in the present study was 0.87 (0.84 men, 0.88 women) for positive dyadic coping and 0.65 (0.64 men, 0.63 women) for negative dyadic coping, which is similar to that found in previous studies. For example, Falconier et al. found an alpha of 0.71 for men and 0.59 for women [34,41].

Depressive symptomatology. Depressive symptoms were measured using the Spanish version [42] of the Center for Epidemiological Studies Depression Scale (CES-D; [43]). This 20-item scale (e.g., “I felt sad”) assesses the frequency of depressive symptoms experienced in the past week. Responses are provided on a 4-point Likert scale, ranging from 0 (rarely or none of the time) to 3 (most or all of the time). Higher scores indicate more frequent depressive symptoms. The internal consistency (Cronbach’s alpha) in the present study was 0.92 (0.92 for men, 0.92 for women).

### 2.3. Data Analysis

**Figure 1:**
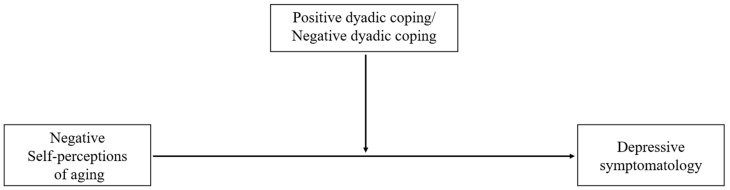
Moderation model. The link from self-perceptions of aging to depressive symptomatology varies as a function of the dyadic coping level (positive dyadic coping or negative dyadic coping).

First, descriptive data and Pearson correlation analyses were conducted. In addition, descriptive characteristics were compared by gender. Second, a simple moderation model (see Figure 1) was tested in which (i) self-perceptions of aging are proposed to influence depressive symptomatology and (ii) the association between self-perceptions of aging and depressive symptomatology is assumed to be moderated by supportive dyadic coping strategies (positive or negative). The moderation effect occurs if the direct association between the independent variable (i.e., self-perceptions of aging) and the dependent variable (i.e., depressive symptomatology) varies across levels of the moderator (positive and negative dyadic coping). Age, education level, years of relationship, having offspring, and physical health were included as covariates.

Prior to model analyses, all predictors and moderators were mean-centered to reduce collinearity between the interaction term and its constituents [44]. To assess gender differences in the moderation model, an interaction term between the moderation and gender was included (self-perceptions of aging x dyadic coping x gender). We included a random intercept at the household level (Level 2) to account for the clustering of couples within a household, since about 28% of the total sample (*n* = 102) involved both individuals from a couple.

Subsequently, the sample was stratified by gender to test the moderation model separately in men and women groups. The models were tested using linear regression analysis. Moreover, post hoc analyses were conducted to test moderation using the PROCESS Macro for SPSS script [45]. PROCESS generated two conditional direct effects: (1) when the moderator (i.e., positive or negative dyadic coping) is at one standard deviation below its mean (low levels of positive or negative DC); and (2) when it is one standard deviation above its mean (high levels of positive or negative DC).

### 2.4. Transparency and Openness

This study was not preregistered. Materials and analysis code for this study are available by emailing the corresponding author.

## 3. Results

### 3.1. Preliminary Analyses

Descriptive data of the main study variables are presented in Table 1 for men, women, and the full sample. Women were younger and reported significantly higher scores in negative dyadic coping and depressive symptomatology and lower scores in positive dyadic coping.

Correlations among the main study variables for men and women are shown in Table 2. As expected, higher levels of depressive symptomatology were associated with more negative self-perceptions of aging and more negative dyadic coping in both men and women. More positive dyadic coping was associated with fewer depressive symptoms only in women.

The moderation model for positive and negative dyadic coping using the whole sample is presented in Table 3. Significant factors included gender, physical health, negative self-perceptions of aging, both positive and negative dyadic coping, and the interaction between self-perceptions of aging and dyadic coping (separately for positive and negative) with gender. The significance of the interaction suggests a gender-dependent difference in the moderation effect. No significant effect of the household variable was found. The proposed models, stratified by gender, are detailed in the following sections.

### 3.2. Moderation Analysis for Positive Dyadic Coping Gender Stratified

The results of the regression analysis for explaining depressive symptoms through the assessed variables, including the product of positive dyadic coping with self-perceptions of aging, are shown in Table 4 for men and women. 

**Figure 2:**
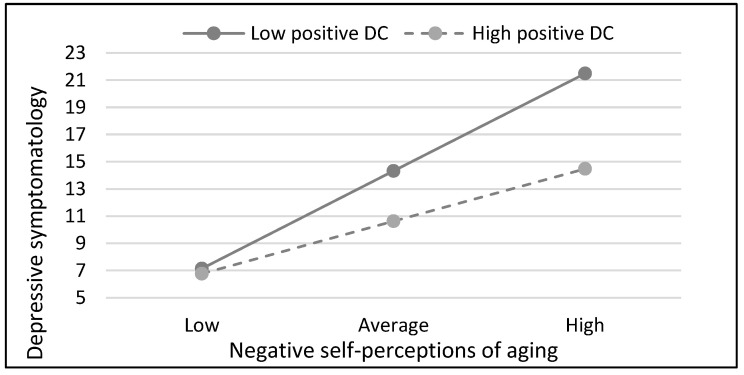
Estimated relationship between negative self-perceptions of aging and depressive symptomatology with low (−1 SD) versus high (+1 SD) positive DC in the group of women.

In the case of women, significant main effects on depressive symptoms were found in the final model for physical health, negative self-perceptions of aging, and positive dyadic coping. In addition to these main effects, a moderator effect was found. That is, the negative self-perception of aging by positive dyadic coping interaction was also significant. Negative self-perceptions of aging were directly and significantly associated with depressive symptomatology regardless of positive dyadic coping level. Nevertheless, the slope of the line showing the association between negative self-perceptions of aging and depressive symptoms is significantly less steep among those with higher perceptions of positive dyadic coping from the partner (see Figure 2). That is, the effect of negative self-perceptions of aging appears to be smaller among those women with higher levels of positive dyadic coping (B = 0.69, SE = 0.8, *p*< 0.001) than among those with lower positive dyadic coping (B = 0.37, SE = 0.08, *p* < 0.001). Among participants with low positive dyadic coping, the mean scores on depression ranged from 7.15 when negative self-perception of aging was low (−1 SD) to 21.50 when it was high (+1 SD). Among participants with high positive dyadic coping, the mean scores on depression raged from 6.77 when negative self-perception of aging was low (−1 SD) to 14.48 when it was high (+1 SD).

For men, significant main effects for the explanation of depressive symptoms were found for age, physical health, and negative self-perceptions of aging. Negative self-perceptions of aging by positive dyadic coping interaction was not significant (*p* = 0.06). The effect of negative self-perceptions of aging on depressive symptomatology did not differ significantly for high or low positive dyadic coping from the partner. That is, positive dyadic coping was not a significant moderator.

### 3.3. Moderation Analysis for Negative Dyadic Coping Gender Stratified

The results of the regression analysis for explaining depressive symptoms through the assessed variables, including the product of negative dyadic coping with self-perceptions of aging, are shown in Table 5 for men and women.

**Figure 3:**
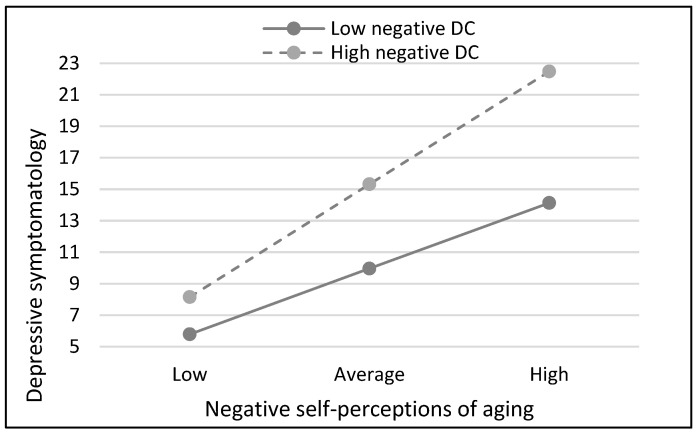
Estimated relationship between negative self-perceptions of aging and depressive symptomatology with low (−1 SD) versus high (+1 SD) negative DC in the group of women.

For women, as shown in Table 5, significant effects of physical health, negative self-perceptions of aging, and negative dyadic coping were found in the final model. In addition to these main effects, a moderator effect was found. That is, the negative self-perception of aging by negative dyadic coping interaction was also significant. This negative self-perception of aging by negative dyadic coping interaction is shown in Figure 3. Negative self-perceptions of aging were directly and significantly associated with depressive symptomatology regardless of negative dyadic coping level. However, the slope of the line showing the association between negative self-perceptions of aging and depressive symptoms is significantly steeper among those with higher perceptions of negative dyadic coping from the partner. That is, the effect of negative self-perceptions of aging appears to be larger among those women with higher levels of negative dyadic coping (B = 0.69, SE = 0.08, *p* < 0.001) than among those with lower negative dyadic coping (B = 0.40, SE = 0.08, *p*< 0.001). Among participants with high negative dyadic coping, the mean scores on depression ranged from 8.16 when negative self-perceptions of aging were low (−1 SD) to 22.48 when it was high (+1 SD). Among participants with low negative dyadic coping, the mean scores on depression ranged from 5.80 when negative self-perceptions of aging were low (−1 SD) to 14.14 when it was high (+1 SD).

In men, a significant effect of age, physical health, and negative self-perceptions of aging was found in the final model for explaining depressive symptoms. Men perceiving high negative dyadic coping reported more depressive symptoms across all levels of negative self-perceptions of aging. Nonetheless, significant negative dyadic coping was not a significant (*p* = 0.15) moderator in the group of men.

## 4. Discussion

Research highlights the need to understand the deleterious effects that negative self-perceptions of aging can have on the quality of life [e.g., 5] and, specifically, the need to understand what factors can help to buffer the effects of negative perceptions of aging. The Systemic Transactional Model (STM; [22,25]) hypothesizes that a close relationship may influence the relationship between stress reactions and outcomes. We aimed to understand how the different types of partner support (negative and positive) may moderate the effect of negative self-perceptions of aging on depressive symptoms. To our knowledge, this is the first study to analyze the effect of the positive and negative supportive DC in the association between negative self-perceptions of aging and depressive symptoms.

Supporting our first hypothesis, and consistent with previous studies [5,6], the obtained findings suggest that regardless of gender, higher levels of negative self-perceptions of aging were significantly associated with higher depressive symptoms. A potential explanation for this finding may be that self-perceptions of aging can function as triggers for passive or avoidant behaviors and thoughts (e.g., avoiding social activities, attributing mood changes to aging) which, although negatively reinforced in the short term by reducing discomfort, lead to maladaptive outcomes in the long term (e.g., social isolation, a perceived loss of control over emotional well-being). These outcomes, in turn, reinforce negative self-perceptions of aging and contribute to greater depressive symptoms [1,2,3,4]. Diverse studies have found that people with more negative self-perceptions of aging might have a more negative view of the future, lower perceptions of control, higher perceived loneliness, poor social engagement, more risky health behaviors, higher reaction to stress and less social contact [2,5,7,13,46,47].

Interestingly, chronological age was not associated with the level of self-perceptions of aging (see Supplementary Materials), which is coherent with what has already been reported in previous studies [6,7]. Also consistent with previous research [34,46,48,49], women in our sample had higher levels of depressive symptomatology and perceived more negative support and less positive support from their partners.

Focusing on DC, in this study, it was assessed by only one member of the couple and was associated with different variables depending on gender. These differences may suggest that the effect of DC on quality of life operates through different mechanisms for men and women. Positive and negative DC were associated with negative self-perceptions of aging in men but not in women. However, in the case of depressive symptoms, positive and negative DC were associated with depressive symptoms in women, whereas only negative DC showed a significant association with depressive symptoms in men.

Even though a significant association between negative self-perceptions of aging and depressive symptomatology is observed regardless of the level of positive and negative DC, the obtained findings suggest that the relationship between negative self-perceptions of aging and depressive symptoms is moderated by positive and negative DC. The moderation effect was moderated by gender. Specifically, perceiving positive DC was associated with fewer depressive symptoms only in women. In addition, positive dyadic coping from the partner may mitigate the potential impact of negative self-perceptions of aging on depressive symptoms. This moderation effect is also reflected in the clinical significance of the findings: among women who showed high levels of negative self-perceptions of aging, those with high positive DC showed an average depression level (mean = 14.48) below the cut-off point on the CES-D scale for probable clinical depression (cut off score < 16); conversely, those who perceived low positive DC had, on average, a CES-D mean score of 21.50, above the cut-off point for probable clinical depression (cut-off score ≥ 16). No significant moderation effect was found for men.

Likewise, negative DC was associated with more depressive symptoms only in women. In addition, negative DC appeared to amplify the association between negative self-perceptions of aging and depressive symptoms. This moderation effect was moderated by gender. In particular, women with high levels of negative self-perceptions and high levels of negative DC had, on average, scores above the cut-off score on the CES-D for clinical depression (mean = 22.48). In the case of women with low levels of negative DC, scores in depressive symptoms were below the cut-off score for clinical depression (mean = 8.16). It seems that when the partner’s support is hostile, superficial, and ambivalent, the effect of negative self-perceptions of aging on depressive symptomatology in women is especially detrimental. Thus, these results support that in women, not perceiving optimal support from their partner when stressed by a negative situation may contribute to feeling even worse about themselves [36].

Therefore, our results indicate that individuals who perceive their partner’s support as positive DC, characterized by empathy, understanding, or providing advice in stressful situations, can be highly beneficial [22,25]. This perception of support may help mitigate potential stress triggered or exacerbated by concerns or challenges related to aging, and they might moderate and reduce the detrimental effects of negative self-stereotypes of aging on depressive symptomatology in women. On the other hand, perceiving support as negative DC by the partner, involving behaviors such as hostility, superficial and ambivalent support provision (e.g., not considering or valuing the stress of the partner or blaming him/her for not coping well with a stressful situation; [22,25]), may increase the stress perceived and then increase the deleterious effect of negative self-perceptions of aging on depressive symptoms.

The results from the present study suggest that positive and negative supportive DC may play a significant role in moderating the relationship between negative aging stereotypes and depressive symptoms, particularly in women. This finding indicates that women may be more sensitive to both positive and negative DC from their partners, which in turn impacts their depressive symptoms. This potential gender difference may be explained by stereotypes related to gender roles, which depict women as more fragile and, hence, more dependent on men [50,51]. However, this explanation might not be sufficient for older women, who are more likely to become caregivers and assert greater social control [30]. In addition to these stereotypes, gender roles also shape emotional expression and coping strategies. Women may be expected to be more emotionally open, able to express and share their feelings, and empathize with others, while men are often socialized to suppress and regulate their emotions [52]. This tendency may result in men being less likely to seek or rely on spousal support, limiting the potential for this support (whether positive or negative) to influence their well-being. Furthermore, previous studies have shown that women are more likely to use social support seeking as a coping strategy [52] even when self-reported depressive symptoms are controlled for [53]. Therefore, future research should explore the reasons behind this difference in older women. The results of the present study can be related to the functioning of DC in response to stressors. Falconier [34] in a study on immigrants found that positive and negative supportive DC was observed to be a moderating variable between stress and well-being only in female participants. Diary-based studies suggest that women may be more attuned to their partner’s positive and negative support [32], indicating that women may pay more attention to their partner’s behavior. Negative self-perceptions of aging may lead women, more than men, to worry about the adequacy and robustness of the care and protection from the partner when actual needs arise [54]. Although dyadic coping seems to play a more important role in women, further studies are needed to explore these gender differences, as several studies have already indicated that DC has an effect on both genders [35,55]. In this sense, Gable et al. [36] found that in both genders, those who perceived greater support from their partners when they reported stressful situations had better partner relationships and well-being than those who perceived low partner support.

The detrimental impact of negative self-perceptions of aging and the beneficial influence of partner relationship quality on depressive symptoms are well known. However, the literature lacked an analysis of the role of positive and negative supportive DC as a moderator in the effect of negative self-perceptions of aging on depressive symptoms of middle-aged and older adults in a partner relationship. Our study is the first to show that when high levels of negative self-perceptions of aging are present, higher positive DC and lower negative DC may act as moderating factors in the relationship with depressive symptoms.

The outcomes associated with negative self-perceptions of aging (e.g., inactivity, social withdrawal, and negative emotions) could be either counteracted or exacerbated by the perception of positive or negative DC from one’s partner. Positive support may mitigate the maladaptive behaviors linked to negative self-perceptions of aging by encouraging engagement in social and physical activities, fostering a sense of emotional security, and reducing feelings of loneliness. In contrast, negative support from a partner may reinforce the avoidant or passive behaviors triggered by negative self-perceptions of aging, potentially worsening the emotional and psychological consequences, such as increased depressive symptoms. Several mechanisms may explain this moderation. First, perceiving marital satisfaction, high positive DC and low negative DC may be associated with higher levels of positive emotions and more satisfaction with life [22,55,56]. Experiencing positive emotions can counteract negative emotions, change habitual modes of thinking, and strengthen personal resources for coping [57]. Optimism and positive emotions seem to be important factors to counter the detrimental implications of negative self-perceptions of aging on depressive symptoms [58]. Second, several studies have reported that individuals with higher levels of negative self-perceptions of aging were less likely to engage in health-promoting activities, which, in turn, resulted in worse physical and mental health [5,59]. Positive support from the partner, on the other hand, increases the engagement in health-related activities, such as exercise [60,61]. In addition, the partner might play an important role in cognitive collaboration to compensate for the natural decline of cognitive skills in later life. Cognitive collaboration may help individuals remain functionally active, since it improves task performance, compensating for the lack of skills, and other functions such as the regulation of relationships and oneself [62]. Finally, a supportive partner may encourage the individual to seek care for any change in health, preventing this being discounted by attributing such changes to the normal course of aging [63,64]. In this sense, declining mental health is more likely if those with negative self-perceptions of aging do not seek health care because they attribute depressive symptoms to normal mental ill-health in the aging process [46,65].

The findings outlined have important clinical implications. Firstly, they highlight the potential benefits of working with partners to address the detrimental effects of negative self-perceptions of aging. Hence, when assessing potential age-related challenges or problems, gerontologists and clinicians should focus on the close relationships of individuals in order to identify potential positive or negative support from their partner. Although women appear to be more vulnerable to the effects of DC by the partner, as this study showed that DC by the partner only significantly moderated the effects in the group of women, these clinical recommendations are valuable for both members of the couple. Men also benefit from adaptive dyadic coping (DC) provided by their partners. As shown in Table 2, it is directly associated with more positive self-perceptions of aging. Additionally, both men’s and women’s perceived engagement in positive dyadic coping efforts predicts their own relationship satisfaction and decreased negative interactions [66]. Furthermore, engagement in negative coping behaviors during times of stress, such as confrontative demand and withdrawal, is associated with negative mood for both genders [67]. Therefore, promoting adaptive dyadic coping strategies can be beneficial for enhancing the well-being and relationship satisfaction of both men and women, ultimately contributing to better mental health and self-perceptions of aging.

Also, on the basis of the mechanisms mentioned previously, and knowing the potential influence that the partner relationship has on the effect of negative self-perceptions of aging on depressive symptoms, some recommendations are proposed. In the first instance, cognitive–behavioral therapy (CBT) to address depressive symptoms in older adults emphasizes the importance of challenging the opinions and stereotypical views of those around the older person [68]. Additionally, individual interventions that address age-related attributions to promote physical activity in older sedentary adults could be enhanced if carried out together with their partners [60]. Therefore, for individuals in a partner relationship, an intervention based on a successful aging model can be more efficient if carried out with the partner and integrating an interpersonal view [69]. Educating about successful aging as a couple can be very beneficial, since typically both partners tend to have similar levels of negative self-perceptions of aging, and stereotypes of one partner are related to negative self-perceptions regarding the future of the other [70]. Greater knowledge of successful aging will allow for better dyadic support to cope with stressful situations. Supportive DC should be considered as a resource when conducting therapies aimed at preventing and reducing negative self-perceptions of aging [4,68]. Interventions with couples might generate a more positive environment or atmosphere at home to motivate both members of the couple to be more active and keep social contact, despite their negative self-perceptions of aging, thus contributing to maintaining their physical and emotional well-being [70,71].

In addition to the significant insight that the analysis of our data provided, we must discuss some limitations of this study. An important caveat is that because of the cross-sectional design, our analyses cannot confirm any causal relationship between the analyzed variables. The associations may have alternative directions, including the possibility that negative self-perceptions of aging moderate the association of positive or negative supportive DC with depressive symptomatology [54]. Furthermore, individuals with more depressive symptoms report more negative self-perceptions of aging [23,26], and they may also perceive and rate DC more negatively [72]. Longitudinal and experimental studies are needed in order to advance our knowledge regarding the causal relationships between the variables. Additionally, longitudinal analyses are also necessary to confirm the differences found based on gender [23]. Regarding the moderation model, future studies could explore more complex models that consider multiple types of dyadic coping (positive, negative, etc.) as simultaneous moderators. Although our sample size was large enough to permit robust statistical analyses, we used a convenience sample, consisting of middle-aged and older adults volunteering for the study. The participants might arguably be skewed toward better functioning individuals and those who had an open attitude to the research question. The sample may therefore not be representative of the general sample of middle-aged people. It is important to note that dyadic coping was assessed mostly in one member of the couple, so we could not evaluate inter-dynamics between partners. In addition, although previous studies have also found a relatively low Cronbach’s alpha for the negative DC subscale, the low Cronbach’s alpha (0.65) obtained for negative DC suggests caution when interpreting the results, making future studies that replicate those obtained here necessary. Moreover, it should be noticed that the mean age was significantly low, so it may be that participants were still in work and maintaining greater social contact, decreasing the importance of partner contact in their emotional regulation [73,74].

## 5. Conclusions

Negative self-perceptions of aging are associated with depression [5], and one explanation of this association may be that individuals with negative aging stereotypes are more susceptible to stress [13]. As many people age in partner relationships, there is growing evidence to suggest that dyadic coping has an important buffering effect on stressors and relationship outcomes [31,34]. Our study adds to previous research by showing that positive and negative supportive DC play an important role in moderating the effect of negative self-perceptions of aging to depressive symptoms in the group of women. Positive and negative DC do not appear to be as significant for explaining depressive symptoms for men as they are for women. When women hold greater levels of negative self-perceptions of aging, they are more likely to experience lower depressive symptoms if they are also perceiving more spousal support (more positive DC and less negative DC). The findings showed that the moderating factors operated more clearly when negative self-perceptions of aging were high. Hence, partner support plays an important role as a protective factor, since clinical depression is observed when negative self-perceptions of aging are high and levels of positive DC are low or negative DC is high. These findings are particularly relevant given the circumstances of older adults for whom their partner is often their main source of social support [25,74].

## Figures and Tables

**Table 1:** Descriptive data of the main study variables.

	Men (*n* = 148)	Women (*n* = 217)		Full Sample	
Variables	M (SD) or Percentage	M (SD) or Percentage	*t*	Range	M (SD) or Percentage
Age	63.72 (9.92)	58.89 (10.73)	4.36 **	40−90	60.86 (10.66)
Level of education ^a^	4.46 (1.68)	4.40 (1.60)	0.34	1−7	4.42 (1.63)
Years of relationship	33.03 (14.57)	30.77 (14.58)	1.46	1−70	31.69 (14.60)
Having offspring	93%	91%			92%
Physical health ^b^	12.33 (3.54)	12.57 (3.09)	−0.68	10−30	12.47 (3.28)
Negative self-perceptions of aging	29.53 (10.97)	30.00 (10.37)	−0.42	12−66	29.71 (17.70)
Positive dyadic coping	19.70 (3.98)	17.77 (4.66)	4.25 **	5−25	10.88 (10.71)
Negative dyadic coping	7.56 (2.95)	8.88 (3.27)	−3.93 **	4−20	29.81 (10.60)
Depressive symptomatology	8.40 (10.08)	12.58 (10.82)	−3.78 **	0−57	18.56 (4.49)

^a^ Higher scores are related to higher level of education. ^b^ Higher scores are related to poorer physical functioning. ** *p* < 0.01.

**Table 2:** Intercorrelations among study variables.

	1	2	3	4
1. Negative self-perceptions of aging		−0.06	−0.04	0.56 **
2. Positive dyadic coping	−0.27 **		−0.42 **	−0.18 **
3. Negative dyadic coping	0.25 **	−0.27 **		0.21 **
4. Depressive symptomatology	0.54 **	−0.12	0.24 **	

Note. The results for the female sample (*n* = 217) are shown above the diagonal. The results for the male sample (*n* = 148) are shown below the diagonal. ** *p* < 0.01.

**Table 3:** Moderation analyses of dyadic coping on the relationship between negative self-perceptions of aging and depressive symptomatology.

	Depressive Symptomatology			
	Model 1		Model 2	
	*b*	*(SE)*	*b*	*(SE)*
Fixed effects				
Intercept	4.35	(4.63)	6.16	(4.60)
Gender	2.92 *	(0.96	2.74 *	(0.94)
Age	−0.09	(0.06)	−0.09	(0.06)
Level of education ^a^	−0.13	(0.30)	−0.20	(0.30)
Years of relationship	−0.02	(0.05)	−0.01	(0.05)
Offspring (1 = yes, 2 = no)	0.08	(1.74)	0.04	(1.72)
Physical health ^b^	0.66 *	(0.15)	0.58 *	(0.15)
Negative self-perceptions of aging	0.45 *	(0.05)	0.46 *	(0.04)
Positive dyadic coping	−0.25 *	(0.10)		
Negative self-perceptions of aging x Positive dyadic coping x gender	−0.02 *	(0.01)		
Negative dyadic coping			0.63 *	(0.14)
Negative self-perceptions of aging x Negative dyadic coping x gender			0.02 *	(0.01)
Random effects				
Intercept variance (L2: Household)	1.29	(9.73)	3.55	(9.64)
Residual variance	69.30 *	(10.96)	65.19 *	(10.66)
2 log likelihood	2579.759		2589.568	

Note: * *p* < 0.001. ^a^ Higher scores are related to higher level of education. ^b^ Higher scores are related to poorer physical functioning.

**Table 4:** Moderation analyses of positive dyadic coping on the relationship between negative self-perceptions of aging and depressive symptomatology (depression as dependent variable).

	Depressive Symptomatology					
	Men			Women		
	Standard Coefficient			Standard Coefficient		
	*B*	*t*	*p*	*B*	*t*	*p*
1. Age	**−0.32**	**−3.44**	**<0.001**	0.07	0.85	0.40
2. Level of education ^a^	−0.08	−1.14	0.26	0.02	0.38	0.70
3. Years of relationship	0.10	1.11	0.27	−0.12	−1.41	0.16
4. Having offspring	0.11	1.45	0.15	−0.04	−0.76	0.45
5. Physical health ^b^	**0.29**	**3.50**	**<0.001**	**0.17**	**2.82**	**0.01**
6. Negative self-perceptions of aging	**0.37**	**4.51**	**<0.001**	**0.51**	**8.87**	**<0.001**
7. Positive dyadic coping	0.00	−0.06	0.96	**−0.17**	**−3.12**	**<0.001**
8. Negative self-perceptions of aging x positive dyadic coping	−0.14	−1.93	0.06	**−0.16**	**−2.92**	**<0.001**
**R^2^**	**39%**			**39.3%**		

Note: ^a^ Higher scores are related to higher level of education. ^b^ Higher scores are related to poorer physical functioning.

**Table 5:** Moderation analyses of negative dyadic coping on the relationship between negative self-perceptions of aging and depressive symptomatology (depression as dependent variable).

	Depressive Symptomatology					
	Men			Women		
	Standard Coefficient			Standard Coefficient		
	*B*	*t*	*p*	*B*	*t*	*p*
1. Age	**−0.32**	**−3.44**	**<0.001**	0.06	0.73	0.47
2. Level of education ^a^	−0.10	−1.42	0.16	0.02	0.28	0.78
3. Years of relationship	0.11	1.20	0.23	−0.11	−1.27	0.21
4. Having offspring	0.09	1.28	0.20	−0.04	−0.62	0.53
5. Physical health ^b^	**0.24**	**3.09**	**<0.001**	**0.17**	**2.85**	**<0.001**
6. Negative self-perceptions of aging	**0.39**	**4.91**	**<0.001**	**0.52**	**9.33**	**<0.001**
7. Negative dyadic coping	0.10	1.46	0.15	**0.25**	**4.65**	**<0.001**
8. Negative self-perceptions of aging x negative dyadic coping	0.10	1.44	0.15	**0.13**	**2.52**	**0.01**
**R^2^**	**39.2%**			**41.7%**		

Note: ^a^ Higher scores are related to higher level of education. ^b^ Higher scores are related to poorer physical functioning.

## Data Availability

Data supporting the findings of this study are available upon request by emailing the corresponding author. Due to privacy and ethical considerations, the dataset is not publicly accessible. Author note: Preliminary and partial data of this study were presented at the 2023 IPA International Congress (Lisbon, 2023). This study was not preregistered. Materials and analysis code for this study are available by emailing the corresponding author.

## Supplementary Materials

The following supporting information can be downloaded at: https://www.mdpi.com/article/10.3390/geriatrics9060147/s1, Table S1: Descriptive data of people who filled the questionary on-line and in person, Table S2:Moderation analyses of positive dyadic coping on the relationship between negative self-perceptions of aging and depressive symptomatology (depression as dependent variable), Table S3: Moderation analyses of negative dyadic coping on the relationship between negative self-perceptions of aging and depressive symptomatology (depression as dependent variable), Table S4: Descriptive and correlation among study variables, Table S5: Moderation Analyses of positive dyadic coping on the relationship between negative self-perceptions of aging and depressive symptomatology, Table S6: Moderation analyses of negative dyadic coping on the relationship between negative self-perceptions of aging and depressive symptomatology.
