# Supplementary material for: Dyadic Coping in Aging: Linking Self-Perceptions of Aging to Depression
Source: Geriatrics (Basel). 2024 Nov 11;9(6):147. doi: 10.3390/geriatrics9060147 (PMC11586940; doi:10.3390/geriatrics9060147)
Supplement: Supplementary file 1 [file geriatrics-09-00147-s001.zip › geriatrics-3251401-supplementary.pdf]

Supplementary information

Characteristics of Data Collection Methods.

Significant differences were found in most variables between the online and face-to-face interview groups. In the control variables, the group that responded online was younger, had a higher level of education, fewer years of relationship, a higher percentage of people with offspring, and better physical health. Regarding the main variables, the group that responded online had more negative self-perceptions of aging, higher negative DC, and more depressive symptoms. However, no differences were found based on gender or positive DC. It cannot be assumed that the response format is a determinant in the main variables since these differences could be attributed to differences in demographic variables or other control variables.

Supplementary Table 1. Descriptive data of people who filled the questionary on-line and in person.

	**on-line (n=248)**	**in person (n=118)**		
**Variables**	**M (SD)**	**M (SD)**	X^2	***t***
Women	61.7%	54.2%	1.842	
Age	58.10 (10.63)	66.67 (8.12)		-8.51**
Level of education [a]	5.00 (1.52)	3.23 (1.12)		12.51**
Years of relationship	28,11 (13.97)	39.21 (12,98)		-7.46**
Having Offspring	88.7%	99.2%	11.95**	
Physical health [b]	12.21 (2.98)	13.02 (3.77)		-2.21*
Negative self-perceptions of aging	31.51 (10.78)	26.24 (9.30)		4.57**
Positive dyadic coping	18.32 (4.41)	19.07 (4.64)		-1.49
Negative dyadic coping	8.67 (3.10)	7.66 (3.33)		2,84**
Depressive symptomatology	12.09 (11.40)	8.33 (8.58)		3,51**

[a] Higher scores are related to higher level of education. [b] Higher scores are related to poorer physical functioning. * $p < .05$ ** $p < .01$

In addition, to control the method effect that the data collection process could have had, we conducted analyses to control for the 'response format' (online or physical) as a covariate. The variable response format was included in the two models in men and women separately. The response format was not a significant predictor for any of the models, which showed the same significances that were found without controlling for this variable (Table S2 and Table S3).

Supplementary Table 2. Moderation analyses of positive dyadic coping on the relationship between negative self-perceptions of aging and depressive symptomatology (depression as dependent variable).

	**Depressive symptomatology**					
	Men			Women		
	Standard coefficient *B*	*t*	*p*	Standard coefficient *B*	*t*	*p*
1. Age	-0.33	-3.46	<.001	0.08	1.01	0.31
2. Level of education [a]	-0.06	-0.77	0.44	-0.02	-0.36	0.72
3. Years of relationship	0.10	1.11	0.27	-0.12	-1.35	0.18
4. Having offspring	0.11	1.45	0.15	-0.05	-0.84	0.40
5. Physical health [b]	0.28	3.46	<.001	0.18	2.92	<.001
6. Negative self-perceptions of aging	0.38	4.50	<.001	0.48	8.16	<.001

7. Positive dyadic coping	-0.01	-0.12	0.91	-0.17	-3.12	<.001
8. Negative self-perceptions of aging x positive dyadic coping	-0.14	-1.96	0.05	-0.16	-3.01	<.001
9. Response format	0.04	0.49	0.62	-0.11	-1.69	0.09
R^2	**39.1%**			**40.1%**		

Note: [a] Higher scores are related to higher level of education.

[b] Higher scores are related to poorer physical functioning.

Supplementary Table 3. Moderation analyses of negative dyadic coping on the relationship between negative self-perceptions of aging and depressive symptomatology (depression as dependent variable).

	**Depressive symptomatology**					
	Men			Women		
	Standard coefficient *B*	*t*	*p*	Standard coefficient *B*	*t*	*p*
1. Age	-0.33	-3.44	<.001	0.07	0.81	0.42
2. Level of education [a]	-0.09	-1.10	0.27	0.00	-0.04	0.97
3. Years of relationship	0.11	1.19	0.24	-0.11	-1.25	0.21
4. Having offspring	0.09	1.27	0.20	-0.04	-0.65	0.51
5. Physical health [b]	0.24	3.02	<.001	0.17	2.87	<.001

6. Negative self-perceptions of aging	0.39	4.85	<.001	0.51	8.78	<.001
7. Negative dyadic coping	0.11	1.50	0.14	0.24	4.48	<.001
8. Negative self-perceptions of aging x negative dyadic coping	0.09	1.38	0.17	0.13	2.43	0.02
9. Response format	0.03	0.34	0.74	-0.05	-0.70	0.49
R^2	**39.2%**			**41.8%**		

Note: [a] Higher scores are related to higher level of education.

[b] Higher scores are related to poorer physical functioning.

Supplementary Table 4.

Descriptive and correlation among study variables

	1	2	3	4	5	6	7	8	9
1. Age		-.35**	.73**	-.24**	.33**	-.11	-.06	.04	.00
2. Level of education [a]	-.32**		-.44**	.19**	-.13	.01	.06	.01	.02
3. Years of relationship	.66**	-.40**		-.39**	.21**	-.12	-.11	.03	-.08
4. Offspring (1=yes, 2=no)	-.20*	.23**	-.25**		-.03	-.02	.03	-.02	-.02
5. Physical health [b]	.34**	-.16	.20*	-.05		.24**	.00	-.02	.29**
6. Negative self-perceptions of aging	-.02	.01	.01	.27**	.40**		-.06	-.04	.56**
7.Positive dyadic coping	-.01	-.01	-.03	-.01	.09	-.27**		-.43**	-.18**
8.Negative dyadic coping	.06	.01	.07	.03	.20*	.25**	-.27**		.21**

9.Depressive symptomatology	-.15	-.05	-.03	.21**	.35**	.54**	-.12	.24**

Note. The results for the female sample (n = 217) are shown above the diagonal. The results for the male sample (n = 148) are shown below the diagonal

**p* < .05, ** *p*< .01

[a] Higher scores are related to higher level of education.

[b] Higher scores are related to poorer physical functioning.

The next two tables represent post hoc analyses conducted to test moderation using the PROCESS Macro for SPSS script. In these tables, the effects of different levels (low and high) of positive or negative dyadic coping can be found.

Supplementary Table 5.

Moderation Analyses of **positive** dyadic coping on the relationship between negative self-perceptions of aging and depressive symptomatology.

Model Summary	Depressive symptomatology (Men)					Depressive symptomatology (Women)				
	Estimate	SE	95% CI		p	Estimate	SE	95% CI		p
			LL	UL				LL	UL	
1. Gender (0=male, 1=female)										
2. Age	**-0.330**	0.096	-0.519	-0.141	0.001	0.071	0.083	-0.094	0.235	0.398
3. Level of education [a]	-0.507	0.445	-1.387	0.374	0.257	0.157	0.408	-0.648	0.962	0.702
4. Years of relationship	0.071	0.064	-0.056	0.197	0.270	-0.091	0.064	-0.217	0.036	0.161
5. Offspring (1=yes, 2=no)	4.263	2.935	-1.539	10.065	0.149	-1.706	2.259	-6.160	2.748	0.451

**6. Physical health [b]**	**0.816**	0.233	0.356	1.277	0.001	**0.595**	0.211	0.179	1.012	0.005
**8. Negative self-perceptions of aging**	**0.341**	0.076	0.191	0.491	0.000	**0.532**	0.060	0.414	0.650	0.000
**9. Positive dyadic coping**	-0.011	0.188	-0.381	0.360	0.956	**-0.396**	0.127	-0.646	-0.146	0.002
**10. Negative self-perceptions of aging x positive dyadic coping**	-0.027	0.014	-0.055	0.001	0.056	**-0.034**	0.012	-0.058	-0.011	0.004
Effects with different levels of positive dyadic coping										
**low**						**0. 6921**	0.083	0.528	0.856	0.000
**high**						**0. 372**	0.079	0.215	0.529	0.000
R^2	**39.19**					**39.30**				

Note: First step (negative self-perception regression) is common to all two models. CI = confidence interval. LL = lower limit. UL = Upper limit. Unstandardized coefficients are reported, bias corrected 95% CI, 10,000 bootstrap samples.

Supplementary Table 6.

Moderation analyses of **negative** dyadic coping on the relationship between negative self-perceptions of aging and depressive symptomatology

Model Summary	Depressive symptomatology (Men)					Depressive symptomatology (Women)				
	Estimate	SE	95% CI		p	Estimate	SE	95% CI		p
			LL	UL				LL	UL	
1. Gender (0=male, 1=female)										
2. Age	**-0.331**	0.096	-0.521	-0.141	0.001	0.060	0.082	-0.101	0.221	0.465
3. Level of education [a]	-0.627	0.443	-1.502	0.249	0.159	0.114	0.401	-0.676	0.904	0.776
4. Years of relationship	0.077	0.064	-0.050	0.205	0.233	-0.080	0.063	-0.204	0.044	0.206
5. Offspring (1=yes, 2=no)	3.751	2.931	-2.045	9.546	0.203	-1.384	2.216	-5.752	2.984	0.533
**6. Physical health [b]**	**0.697**	0.226	0.251	1.144	0.002	0.589	**0.207**	0.181	0.997	0.005
**8. Negative self-perceptions of aging**	**0.356**	0.073	0.213	0.500	0.000	0.547	**0.059**	0.431	0.662	0.000
**11. Negative dyadic coping**	0.346	0.236	-0.121	0.813	0.145	0.819	**0.176**	0.472	1.166	0.000
12. Negative self-perceptions of aging x negative dyadic coping	0.033	0.023	-0.012	0.077	0.153	0.044	**0.018**	0.010	0.079	0.013
Effects with different levels of negative dyadic coping										
**low**						**0.402**	0.082	0.240	0.564	0.000

**high**		**0.691**	0.082	0.530	0.852	0.000
R^2	**39.19**	**41.68**				

Note: First step (negative self-perceptions regression) is common to all two models. CI = confidence interval. LL = lower limit. UL = Upper limit. Unstandardized coefficients are reported, bias corrected 95% CI, 10,000 bootstrap samples
